# Post-Operative Pneumonia

**Published:** 1904-06-18

**Authors:** 


### POST-OPERATIVE PNEUMONIA.

Some suggestive experiments bearing on post-operative pneumonia are recorded by Dr. W. L. Chapman. On account of the frequency with which the condition is attributed to the effects of the anæsthetic, and especially so if ether has been used, Dr. Chapman[1] directed his attention towards this agent. He first observed the local effect produced by applying ether to the web of a frog's foot; he then compared macroscopically and microscopically the condition of the lungs of a normal rabbit with that found in the lungs of (*a*) a rabbit which had been etherised, (*b*) a rabbit which had been made to respire an atmosphere laden with Fraenkel's diplococcus pneumoniæ, (*c*) a rabbit which had been both etherised and made to inspire Fraenkel's diplococcus.

When ether was applied, either in the liquid or gaseous condition, to the web of a frog's foot, it produced intense congestion and complete stasis of blood, and in some places small hæmorrhages occurred. After five minutes' exposure of the foot to ether vapour, the circulation was not completely restored until nearly an hour had passed. If the exposure was continued for 10 minutes, the circulation was entirely destroyed and the tissues were killed. These phenomena were not due to cold, Dr. Chapman states, for ice applied to the frog's foot merely caused a slowing of the blood current without complete stasis. Macroscopically the lungs of a rabbit which had been previously anæsthetised with ether showed superficial hæmorrhages. Microscopically the alveoli were seen to be filled with corpuscles, while there was marked congestion of the bronchial tissue. In some instances intra-alveolar hæmorrhages were observed. The changes found were most marked after prolonged anæsthesia, while very brief etherisation caused but little change. To learn if a rabbit previously etherised would acquire pneumonia, one was placed under ether for half an hour on three successive days, and in the intervals it was made to breath an atmosphere laden with Fraenkel's diplococcus. Pneumonia occurred in this animal, while a control rabbit which was not etherised but was made to inspire the same pneumococcus-laden atmosphere remained free from pneumonia. As a result of these experiments Dr. Chapman draws the following conclusions :—(1) Care must be taken by avoiding the use of an unnecessarily large quantity of ether to cause as little respiratory irritation as possible. (2) The disinfection of the mouth and oropharynx before an operation is a rational precaution. (3) Adequate air space is of even greater importance in surgical wards than in medical wards. (4) The chest must be carefully examined before every operation, and if signs of disease be present the operation should be postponed if possible.

Although Dr. Chapman's experiments do not appear to have been sufficiently numerous to afford a perfectly secure basis for future guidance, they are at least suggestive, and it is to be hoped that an early opportunity will be taken to follow up the inquiry along similar lines.

[1] Annals of Surgery, May 1904.

